# ANGIOGENES: knowledge database for protein-coding and noncoding RNA genes in endothelial cells

**DOI:** 10.1038/srep32475

**Published:** 2016-09-01

**Authors:** Raphael Müller, Tyler Weirick, David John, Giuseppe Militello, Wei Chen, Stefanie Dimmeler, Shizuka Uchida

**Affiliations:** 1 Institute of Cardiovascular Regeneration, Centre for Molecular Medicine, Goethe University Frankfurt, Theodor-Stern-Kai 7, Frankfurt am Main 60590, Germany; 2 German Center for Cardiovascular Research, Partner side Rhein-Main, Frankfurt am Main 60590, Germany; 3 THM - University of Applied Sciences, Department MNI, Wiesenstr. 14, D-35390 Giessen, Germany; 4 Laboratory for Functional and Medical Genomics, Berlin Institute for Medical Systems Biology, Lindenberger Weg 80, Berlin 13125, Germany; 5 German Center for Cardiovascular Research, Partner side Berlin, Berlin 13125, Germany; 6 Department of Biology, South University of Science and Technology of China, 1088 Xueyuan Rd, Nanshan District, Shenzhen, Guangdong 518055, China

## Abstract

Increasing evidence indicates the presence of long noncoding RNAs (lncRNAs) is specific to various cell types. Although lncRNAs are speculated to be more numerous than protein-coding genes, the annotations of lncRNAs remain primitive due to the lack of well-structured schemes for their identification and description. Here, we introduce a new knowledge database “ANGIOGENES” (http://angiogenes.uni-frankfurt.de) to allow for *in silico* screening of protein-coding genes and lncRNAs expressed in various types of endothelial cells, which are present in all tissues. Using the latest annotations of protein-coding genes and lncRNAs, publicly-available RNA-seq data was analyzed to identify transcripts that are expressed in endothelial cells of human, mouse and zebrafish. The analyzed data were incorporated into ANGIOGENES to provide a one-stop-shop for transcriptomics data to facilitate further biological validation. ANGIOGENES is an intuitive and easy-to-use database to allow *in silico* screening of expressed, enriched and/or specific endothelial transcripts under various conditions. We anticipate that ANGIOGENES serves as a starting point for functional studies to elucidate the roles of protein-coding genes and lncRNAs in angiogenesis.

With the advent of next generation sequencing techniques, especially RNA sequencing (RNA-seq), comprehensive profiling of transcripts is now possible. The accumulating data suggests that protein-coding genes constitutes a minor part of the mammalian genome as most of the genome are transcribed as RNA but not translated to proteins^1^,^2^,^3^. All those transcripts that do not code for proteins are called “non-coding RNAs (ncRNAs)”, which include a new class of ncRNAs named “long noncoding RNAs (lncRNAs)”^4^. Their definition is very broad, including any non-coding transcripts longer than 200 nucleotides (nt). Although an official nomenclature for lncRNAs does not currently exist, they are generally defined based on their genomic positions relative to nearby protein-coding genes. For example, it is speculated that antisense or sense lncRNAs (that is, in the opposite or same strand, respectively, of protein-coding gene) affect the expression of nearby protein-coding genes (*cis*-regulation). Conversely, long intergenic lncRNAs (lincRNAs, distant from protein-coding genes) likely exert their functions in other genomic locations (*trans*-regulation)^5^. However, such speculations are usually based on guilty-by-association studies from high-throughput data (e.g. microarrays, RNA-seq); in reality, the functional studies of lncRNAs are still scarce.

Despite the number of protein-coding genes still not being fixed^6^, the actual number of lncRNAs is a large matter of debate. Current estimations speculate at least twice as many lncRNAs as protein-coding genes. Recent studies by John Mattick and John Rinn’s groups suggest much more lncRNAs exist, but many lncRNAs are difficult to detect due to their very low expression levels compared to protein-coding genes^7^. However, further investigation is needed to determine if these lowly expressed lncRNAs possess biological functions. Since there is high interest to study lncRNAs in the scientific community, a number of databases were set up, such as ALDB^8^, C-It-Loci^9^, ChIPBase^10^, Co-LncRNA^11^, deepBase v2.0^12^, LncBase v.2^13^, Human Body Map lincRNAs^14^, lncRNA2function^15^, lncRNAdb v2.0^16^, lncRNAMap^17^, lncRNAtor^18^, MTD^19^, NONCODE 2016^20^, NRED^21^, TANRIC^22^ and TF2lncRNA^23^. Furthermore, NCBI and ENSEMBL databases also annotate lncRNAs and include them in their databases. Within these publicly available databases, NONCODE v4^24^ contains the most human lncRNAs, with 56,018 lncRNA genes and 95,135 transcripts. Some of these databases also provide expression profiles of lncRNAs extracted from microarrays and/or RNA-seq. Although it is known that lncRNAs are more tissue/cell-type specifically expressed and potentially regulated than protein-coding genes^25^,^26^, there is no database that is designed specifically for one cell type to comprehensively characterize lncRNAs that are expressed in the target cell type. This is especially problematic when researchers would like to study lncRNAs in their favorite cell type as it is of an economic burden if performing RNA-seq experiment is a prerequisite for their study. Given the large amount of freely available RNA-seq data from large-scale international collaborations and from individual laboratories, an efficient, targeted *in silico* screening and data mining of publicly-available RNA-seq data is needed to identify lncRNAs in a particular cell type and to further characterize them so that more directed, functional studies can be conducted without further screening.

Given the above situation in the field of lncRNAs, in this study, we built a knowledge database “ANGIOGENES” (http://angiogenes.uni-frankfurt.de) to facilitate *in silico* screening of transcripts (e.g. protein-coding genes, lncRNAs) that are expressed in endothelial cells. Endothelial cells are found in all tissues in human body as well as any other organisms with the circulation of blood and therefore, likely misrepresented by tissue level specificity studies. By collecting publicly-available RNA-seq data of endothelial cells from human, mouse and zebrafish under various conditions, the data were analyzed in the same manner to allow for the comparison among different endothelial cells based on expression levels of transcripts. To allow for a hypothesis-generating screening, an easy-to-use web interface is provided to make the usage of ANGIOGENES for non-bioinformaticians. We anticipate that ANGIOGENES will serves as a starting point for the further biological study of the functions of protein-coding genes and lncRNAs within endothelial cells.

## Results

### Building and features of ANGIOGENE knowledge database

In order to collect RNA-seq data sets of endothelial cells, various databases, including Gene Expression Omnibus (GEO), PubMed and Sequence Read Archive (SRA), were manually searched. Since the quality of the genomic sequence information and gene annotations varies depending on the organisms, we chose three well-annotated organisms for further study, which are human, mouse and zebrafish. As shown in Supplementary Table S1, endothelial cells from various body parts under different conditions were collected and analyzed as described in the Methods section. The analyzed data sets were imported into MySQL as sketched out in Figure S1.

**Figure 1:**
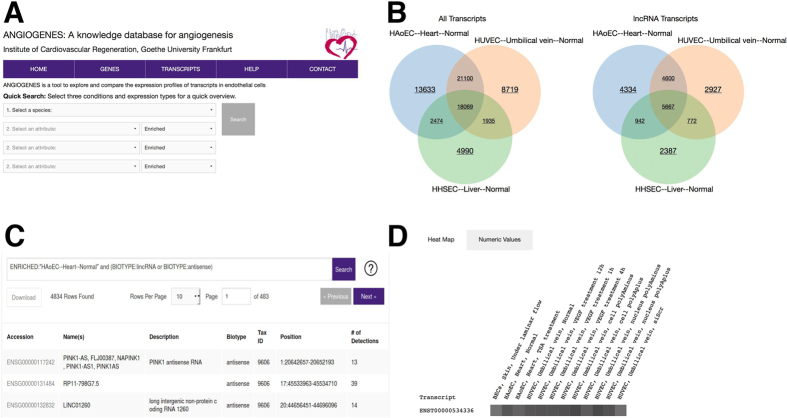
ANGIOGENES web interface. (**A**) Top page of ANGIOGENES. (**B**) Result of a quick search. Each field of a Venn diagram is clickable to provide the list of the corresponding transcripts. (**C**) The result table of transcripts from the selected field of Venn diagram shown in (**B**). The information about each transcript is shown as well as the link to the more detailed information page for each transcript is reachable by clicking on the “Accession” column. (**D**) The heat map and the corresponding expression values of the transcript.

To allow for easy usage, a web interface was created, which can be freely accessed without registration. In the top page of ANGIOGENES (Fig. 1A), a quick search function is provided, allowing comparison of 2 or 3 conditions to screen for expressed, enriched or specific transcripts for each condition. The expression types are defined as follows: “Expressed” (Fragments Per Kilobase of exon per Million fragments mapped (FPKM) values above zero), “Enriched” (FPKM values greater than or equal to the average of all tissues for the target transcript) and “Specific” (expressed only in the target tissue). Once conditions and corresponding expression types are selected and the [Search] button is pressed, results will be shown as Venn diagrams for all transcripts and for lncRNA transcripts (Fig. 1B). These Venn diagrams are clickable. When a field in the diagram is clicked, a new window tab will open to display the transcripts categorized under the specified field along with the advanced search query used to select it. The list of transcripts is displayed as a table for the further examination (Fig. 1C). The link is provided for each transcript, which can be initiated by clicking on an ENSEMBL ID shown in the “Accession” column of the result table. When the accession is clicked, the detailed information about the corresponding transcript will be displayed, which includes the links to other databases (e.g. ENSEMBL, AmiGO 2) as well as embedded genome browser to inspect the genomic location of the transcript. Furthermore, an intuitive heat map describing the expression of the corresponding transcript is provided (Fig. 1D). The information shown in the heat map can be further divided based on the types of cell lines, tissues and conditions, which can be selected through the provided check boxes. The actual FPKM values can be displayed by clicking on the “Numeric Values” tab placed next to the “Heat Map” tab. In order to provide an easy viewing, the heat map is created using the FPKM values above zero. In other words, only those conditions in which the target transcript is expressed will be shown.

Besides an easy-to-use quick search function, more powerful “advanced search” function is provided, which allows users to filter results using search tags and Boolean operators (“and”, “or”, “not”, and “()”). Together, these functions allow for arbitrarily complex searches, constrained only by the users imagination. Furthermore, a wildcard is allowed to search for related terms. This search function is enabled through the “GENES” and “TRANSCRIPTS” tabs to screen for genes and transcripts, respectively. The detailed description of this search function is provided in the help page of ANGIOGENES.

Given that three organisms are included in ANGIOGENES, homology information among three species are provided based on the ENSEMBL database (which are given in the “Homolog” field) as well as our recently introduced concept of positional conservation^9^. The underlying concept of positional conservation is that a genomic locus spanning between two homologous protein-coding genes are conserved when these protein-coding genes are conserved between/among organisms. By defining this locus to be conserved, any transcripts (e.g. lncRNAs) in this locus are also considered as conserved between/among organisms. Using this concept, evolutionary-conserved long intergenic non-coding RNAs (lincRNAs) can be screened. Furthermore, two more conservation information is included as done before^9^. One is based on the ultraconserved elements, which are species-conserved regions that are shown to be transcriptional regulators of key developmental genes^27^,^28^; and another based on the species-conserved *cis*-regulatory elements (enhancers) that are experimentally validated in transgenic mice^29^,^30^. Using the above conservation information (which are given in the “Conserved Regions” field), a user can explore expression patterns of conserved transcripts across organisms. In order to facility further *in silico* screening, a link out to our previously introduced knowledge database for various tissues called “C-It-Loci”^9^ is provided for each conserved locus.

### Validity of ANGIOGENES

To validate the content of ANGIOGENES, first, the expressed genes for each endothelial cell sample (excluding those treated with any drugs nor isolated from gene knockout organisms) was searched and compared to the known published genes in the PubMed database as previously performed^31^,^32^,^33^. Although a very general search term was used (the search term “endothelial” to PubMed, which resulted in the identifications of 3,631, 1,257 and 4,478 ENSEMBL Gene IDs for human, mouse and zebrafish, respectively; PubMed accessed on September 8, 2015), over 60% coverage was achieved for all conditions and organisms, suggesting the analyzed RNA-seq data included in ANGIOGENES reflects reported and published for endothelial cells (Table 1).

As it is known that the correlation of expression levels between RNAs and proteins is rather low^34^, we tested whether the RNA-seq data included in ANGIOGENES reflects the expressions levels of proteins by surveying the Human Protein Atlas (HPA) database^35^. From the normal tissue data, which were generated based on immunohistochemistry experiments using tissue micro arrays, ENSEMBL Gene IDs that are expressed in “endothelial cells” were screened, which resulted in the identification of 10,925 ENSEMBL Gene IDs (HPA accessed on September 8, 2015). These endothelial-expressed proteins were searched via ANGIOGENES to see whether their expressions can also be recorded at the level of transcriptomics by RNA-seq. As shown in Table 2, in all conditions, the coverage is above 60%, suggesting ANGIOGENES contains most of the protein-coding genes expressed in endothelial cells as well.

**Figure 2:**
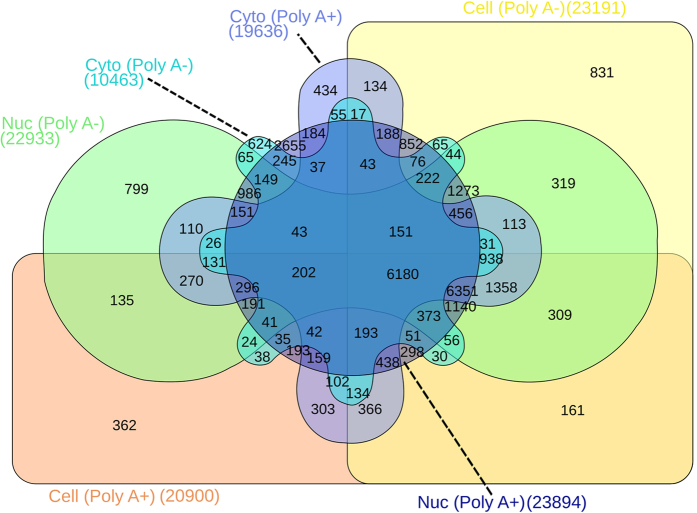
Comparison of HUVEC Transcriptomes. A 6-way Venn diagram represents the numbers of transcripts detected in each preparation of total RNAs with and without poly A tails. “Cell” stands for “whole cell”; “Nuc” for “nucleus”; and “Cyto” for “cytosol”.

Until now, the validation steps performed above mainly concerned protein-coding genes. Given that many lncRNAs have subcellular localizations (e.g. only in the nucleus)^36^ and ~40% of lncRNAs are known not to have poly A tails^37^, we included the long RNA-seq data sets from the ENCODE project, which were generated from RNAs isolated from the nucleus and cytoplasm separately (Supplementary Table S1). Furthermore, RNA-seq libraries were made from poly A + and - fractions separately to better cover the transcriptomes of cell lines, including human umbilical vein endothelial cells (HUVEC). In order to characterize transcriptomes of HUVEC, the following 6 queries were made (Supplementary Table S2): [1] EXPRESSED:“HUVEC–Umbilical vein–cell polyAplus”; [2] EXPRESSED:“HUVEC–Umbilical vein–cell polyAminus”; [3] EXPRESSED:“HUVEC–Umbilical vein–nucleus polyAminus”; [4] EXPRESSED:“HUVEC–Umbilical vein–nucleus polyAplus”; [5] EXPRESSED:“HUVEC–Umbilical vein–cytosol polyAplus”; and [6] EXPRESSED:“HUVEC–Umbilical vein–cytosol polyAminus”. These queries correspond to total RNAs from whole cell with poly A tails, from whole cell without poly A tails, from the nuclear fraction with poly A tails, from the nuclear fraction without poly A tails, from the cytosol fraction with poly A tails, and from the cytosol fraction without poly A tails, respectively. Using the results of these 6 queries, a 6-way Venn diagram was drawn (Fig. 2). By applying Boolean operators to the above queries, it is possible to screen for transcripts that are only detected in the nucleus without Poly A tails and those that are specific to HUVEC but not other cell types under different conditions; which will be (Supplementary Table S2): SPECIFIC:“HUVEC–Umbilical vein–nucleus polyAminus” and not EXPRESSED:“HUVEC–Umbilical vein–cell polyAplus” and not EXPRESSED:“HUVEC–Umbilical vein–cell polyAminus” and not EXPRESSED:“HUVEC–Umbilical vein–nucleus polyAplus” and not EXPRESSED:“HUVEC–Umbilical vein–cytosol polyAplus” and not EXPRESSED:“HUVEC–Umbilical vein–cytosol polyAminus”. This query yields 235 genes. When the following query is added to the previous query, it is possible to restrict the search only for lncRNAs (Supplementary Table S2): (BIOTYPE:lincRNA or BIOTYPE:processed_transcript or BIOTYPE:3prime_overlapping_ncrna or BIOTYPE:antisense or BIOTYPE:non_coding or BIOTYPE:retained_intron or BIOTYPE:sense_intronic or BIOTYPE:sense_overlapping). This combined search resulted in the identification of 59 lncRNAs that are expressed exclusively in the nucleus of HUVEC and do not have poly A tails. With the powerful search function provided in ANGIOGENES, it is possible to screen for a particular set of transcripts, including lncRNAs under various conditions and organisms.

## Discussion

Although it is well known in the field that lncRNAs are cell-type-specifically expressed than protein-coding genes^2^,^3^, until now, there was no database specifically designed for one cell type. To fill this gap in the field, we introduce a knowledge database for endothelial cells called “ANGIOGENES”. ANGIOGENES allows users to screen for various types of transcripts, including protein-coding genes and lncRNAs, within endothelial cells derived from various tissues of human, mouse and zebrafish. Compared to other cell types (e.g. cardiomyocytes, neurons, smooth muscle cells), the isolation and culturing of endothelial cells are more established. In ANGIOGENES, expression profiles of various transcripts whose identifications are based on the ENSEMBL database are included. By incorporating the RNA-seq data sets of endothelial cells isolated from various tissues and/or different conditions, it is possible to perform *in silico* screening of interesting transcripts.

The inclusion of protein-coding genes in ANGIOGENES offers a number of advantages to study lncRNAs. For example, one might be able to find proteins related to lncRNAs (e.g. lncRNA that might regulate protein-coding genes in *cis*) by returning only nearby lncRNAs expressed when the protein is expressed or not expressed. Furthermore, it is possible to crosscheck with the published results of protein functions in relation to treatments in endothelial cells. For this purpose, the associated GO terms and the link out to the Amigo 2 database are provided. This allows for the validation-by-itself style of confirmation, where identified lncRNAs might have similar biogenesis and/or functions under the particular type of endothelial cells and/or in the particular condition by relying on the guilt-by-association relationship.

Currently, single-cell RNA-seq technique is available. However, it is not of a common method to be employed as the amount of total RNAs that one could isolate from one cell is of in the order of pico-grams^38^; in other words, many cycles of PCR-based amplification is necessary to generate enough material for the library preparation of RNA-seq experiment. Since it is well known that cell-to-cell variability is high even in the cells (e.g. HeLa cells) cultured in one cultural dish^39^, although the information obtained from single-cell RNA-seq data sets is of great interest, we did not include such data sets in ANGIOGENES as it is still difficult to perform functional studies with only one cell. In other words, it would not be feasible to obtain enough biological material to run downstream analyses, such as Western blotting experiments. Since the main objective of ANGIOGENES is to provide a starting stage for the identification of interesting set of transcripts, we only included RNA-seq data generated from bulk isolations of endothelial cells.

As stated in the introduction, currently, there is no lncRNA database focused on individual cell type. Furthermore, when similar databases of endothelial cells and angiogenesis were searched, there only three databases are available: Causal Biological Network database^40^, dbANGIO^41^ and PubAngioGen^42^. All of these databases are based on rather manual literature/text mining methods. Unlike these databases, ANGIOGENES is based on experimental RNA-seq data and contains information about lncRNAs, which is absent in the other databases.

Taken together, ANGIOGENES is the only knowledge database of protein-coding genes and lncRNAs that specifically designed for endothelial cells. We anticipate that the prototype proposed in this study should facilitate the further development of knowledge databases designed for a particular cell type.

## Methods

### RNA-seq assembly

**Figure 3:**
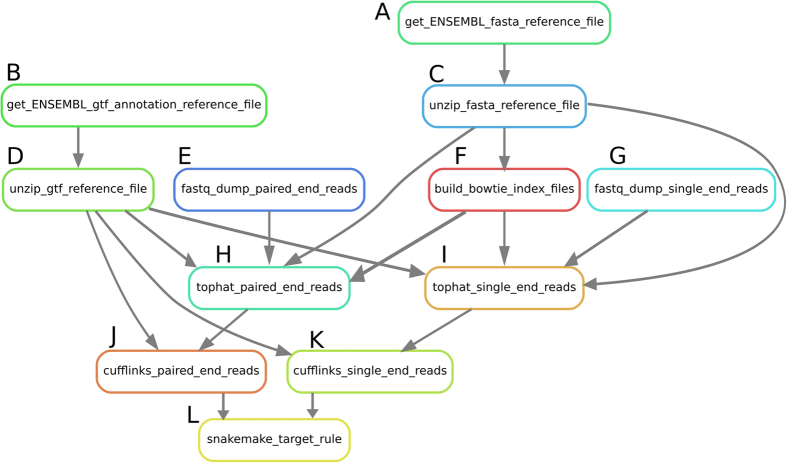
A DAG describing the assembly pipeline used in ANGIOGENES. In this directed acyclic graph (DAG), nodes represent rules, while edges represent transfer of files. Headings (**A**–**L**) contain descriptions of the operations preformed by each node. (**A**) Download the appropriate genome in the FASTA format from ENSEMBL. (**B**) Download the current ENSEMBL annotation. (**C**) Unzip the genome FASTA file. (**D**) Unzip the genome annotations. (**E**) For single-end reads, convert SRA files to FASTQ files. (**F**) Build a Bowtie2 index from the given genome FASTA file. (**G**) For paired-end reads, convert SRA files to FASTQ files. (H) Align single-end reads with Tophat2. (**I**) Align paired-end reads with Tophat2. (**J**) Assemble single-end aligned reads with Cufflinks. (**K**) Assemble paired-end aligned reads with Cufflinks. (**L**) The “snakemake_target_rule” is a special case representing the desired output from the pipeline. In this case, the “transcripts.gtf” files produced by Cufflinks satisfy the rule. More detailed description is provided in the “Snakefile” file at https://bitbucket.org/raistlin91/angiogenes_pipeline/src.

Figure 3 shows a directed acyclic graph (DAG) describing the Snakemake^43^ pipeline used in ANGIOGENES. Specific details regarding the arguments used to run the pipelines can be found in the Snakemake files hosted at https://bitbucket.org/raistlin91/angiogenes_pipeline. Raw sequence data sets were downloaded from the NCBI Sequence Read Archive as SRA files or from EBI as fastq files^44^,^45^. In the case of SRA files, fastq-dump (version 2.1.7) was used to convert the SRA files to fastq files (http://www.ncbi.nlm.nih.gov/sra). The reads were mapped with Tophat (version 2.0.11) and annotated with Cufflinks (version 2.2.1) using the Ensembl GTF annotations; both using the default settings as done previously^9^,^46^,^47^. GRCh38 v77, GRCm38 v81 and GRCz10 v80 genome assemblies and annotations were used for human, mouse and zebrafish, respectively. For the quantification of transcripts, FPKM was used for both single- and pair-end sequences as RPKM (reads per kilobase of exon per million reads) is nearly the same as FPKM. FPKM simply substitutes reads for fragments (i.e. cDNA fragments). Therefore, when using single end reads, FPKM and RPKM are equivalent. However, for paired-end reads, FPKM is not equal to RPKM, as a fragment would normally consist of two reads. Only annotated transcripts with FPKM values greater than 1e-5 were added to the ANGIOGENES database.

### The ANGIOGENES database

All the information and transcript expression values from analyzed RNA-seq data were stored in a MySQL database. The web interface was created using the CakePHP web framework.

Gene Ontology (GO) annotations were obtained from the GO annotations available for each transcript on the Ensembl database. The GO terms available from the gene view are simply the unique set of GO terms for each transcript produced by the gene. For each GO term, a link to the AmiGo 2 database is provided (http://amigo.geneontology.org/amigo)^48^.

The ANGIOGENES database will be updated annually to reflect the current trend of endothelial cells and angiogenesis.

### Validations

To relate the obtained PubMed IDs (PMIDs) to ENSEMBL IDs, the downloaded list of PMIDs for the specific term was linked to ENSEMBL IDs using “gene2pubmed” and “gene2ensembl” from the Entrez Gene database via a custom PERL script. A similar approach was used for the data downloaded from the Human Protein Atlas database^35^. The 6-way Venn diagram was created via InteractinVenn (http://www.interactivenn.net).

## Additional Information

**How to cite this article**: Müller, R. *et al*. ANGIOGENES: knowledge database for protein-coding and noncoding RNA genes in endothelial cells. *Sci. Rep.* **6**, 32475; doi: 10.1038/srep32475 (2016).

## Supplementary Material

Supplementary Information

Supplementary Dataset 1

Supplementary Dataset 2

## Figures and Tables

**Table 1:** Match between ANGIOGENES and PubMed.

Organism	Query Term	# of Matched Genes	# of Genes in PubMed	Coverage (%)
Human	EXPRESSED:“HAoEC–Heart–Normal”	30,771	3,754	83.83
Human	EXPRESSED:“BECs–Skin–Normal”	20,300	3,142	70.17
Human	EXPRESSED:“HUVEC–Umbilical vein–Normal”	35,630	4,022	89.82
Human	EXPRESSED:“HHSEC–Liver–Normal”	20,618	3,020	67.44
Human	EXPRESSED:“HMVEC-D–Skin–Normal”	18,326	3,166	70.70
Mouse	EXPRESSED:“Tie2plus EC–Cerebral cortex–Normal”	18,952	2,993	82.43
Mouse	EXPRESSED:“C166–Yolk sac–Normal”	19,523	2,861	78.79
Mouse	EXPRESSED:“Tie2plus EC–kidney–Normal”	21,923	2,875	79.18
Mouse	EXPRESSED:“mES–Embryoid body–Normal”	18,850	2,674	73.64
Zebrafish	EXPRESSED:“LECs–Lymphatic–FACS isolation of Kaede photconverted red ECs”	13,647	799	63.56

The percent coverage was calculated by dividing the “# of Gene IDs in PubMed” (the match between the query result of ANGIOGENES and Gene IDs listed under “endothelial” in PubMed) by ENSEMBL Gene IDs found associated to the term “endothelial” in PubMed.

**Table 2:** Match between ANGIOGENES and Human Protein Atlas.

Query Term	# of Matched Genes	# of Genes in HPA	Coverage (%)
EXPRESSED:“HAoEC–Heart–Normal”	30,771	9,159	83.84
EXPRESSED:“BECs–Skin–Normal”	20,300	7,552	69.13
EXPRESSED:“HUVEC–Umbilical vein–Normal”	35,630	9,675	88.56
EXPRESSED:“HHSEC–Liver–Normal”	20,618	7,089	64.89
EXPRESSED:“HMVEC-D–Skin–Normal”	18,326	7,861	71.95

The percent coverage was calculated by dividing the “# of Gene IDs in HPA (Human Protein Atlas)” (which is the match between the query result of ANGIOGENES and ENSEMBL Gene IDs listed under “endothelial” in HPA) by ENSEMBL Gene IDs found associated to the term “endothelial” in HPA.
