# Supplementary material for: ANGIOGENES: knowledge database for protein-coding and noncoding RNA genes in endothelial cells
Source: Sci Rep. 2016 Sep 1;6:32475. doi: 10.1038/srep32475 (PMC5007478; doi:10.1038/srep32475)
Supplement: Supplementary Information [file srep32475-s1.doc]

**ANGIOGENES: knowledge database for protein-coding and noncoding RNA genes in endothelial cells**

Raphael Müller^1,2,3*^, Tyler Weirick^1,2*^, David John^1,2*^, Giuseppe Militello^1,2^, Wei Chen^4,5,6^, Stefanie Dimmeler^1,2^, and Shizuka Uchida^1,2^

^1^Institute of Cardiovascular Regeneration, Centre for Molecular Medicine, Goethe University Frankfurt, Theodor-Stern-Kai 7, Frankfurt am Main 60590, Germany

^2^ German Center for Cardiovascular Research, Partner side Rhein-Main, Frankfurt am Main 60590, Germany

^3^ THM - University of Applied Sciences, Department MNI, Wiesenstr. 14, D-35390 Giessen, Germany

^4^Laboratory for Functional and Medical Genomics, Berlin Institute for Medical Systems Biology, Lindenberger Weg 80, Berlin 13125, Germany

^5^German Center for Cardiovascular Research, Partner side Berlin, Berlin 13125, Germany

^6^Department of Biology, South University of Science and Technology of China, 1088 Xueyuan Rd, Nanshan District, Shenzhen, Guangdong 518055, China

*These authors contributed equally to this work.

Correspondence and requests for materials should be addressed to S.U. (email: heart.lncrna@gmail.com)

**Additional Information**

Two tables and one figure are available as supplementary data.

**Supplementary Table S1. List of NGS data sets used for ANGIOGENES.**

**Supplementary Table S2. Links to the queries used in the manuscript.**

**Supplementary Figure S1. Database scheme of ANGIOGENES.**
