# Supplementary figures and images for: ANGIOGENES: knowledge database for protein-coding and noncoding RNA genes in endothelial cells
Source: Sci Rep. 2016 Sep 1;6:32475. doi: 10.1038/srep32475 (PMC5007478; doi:10.1038/srep32475)

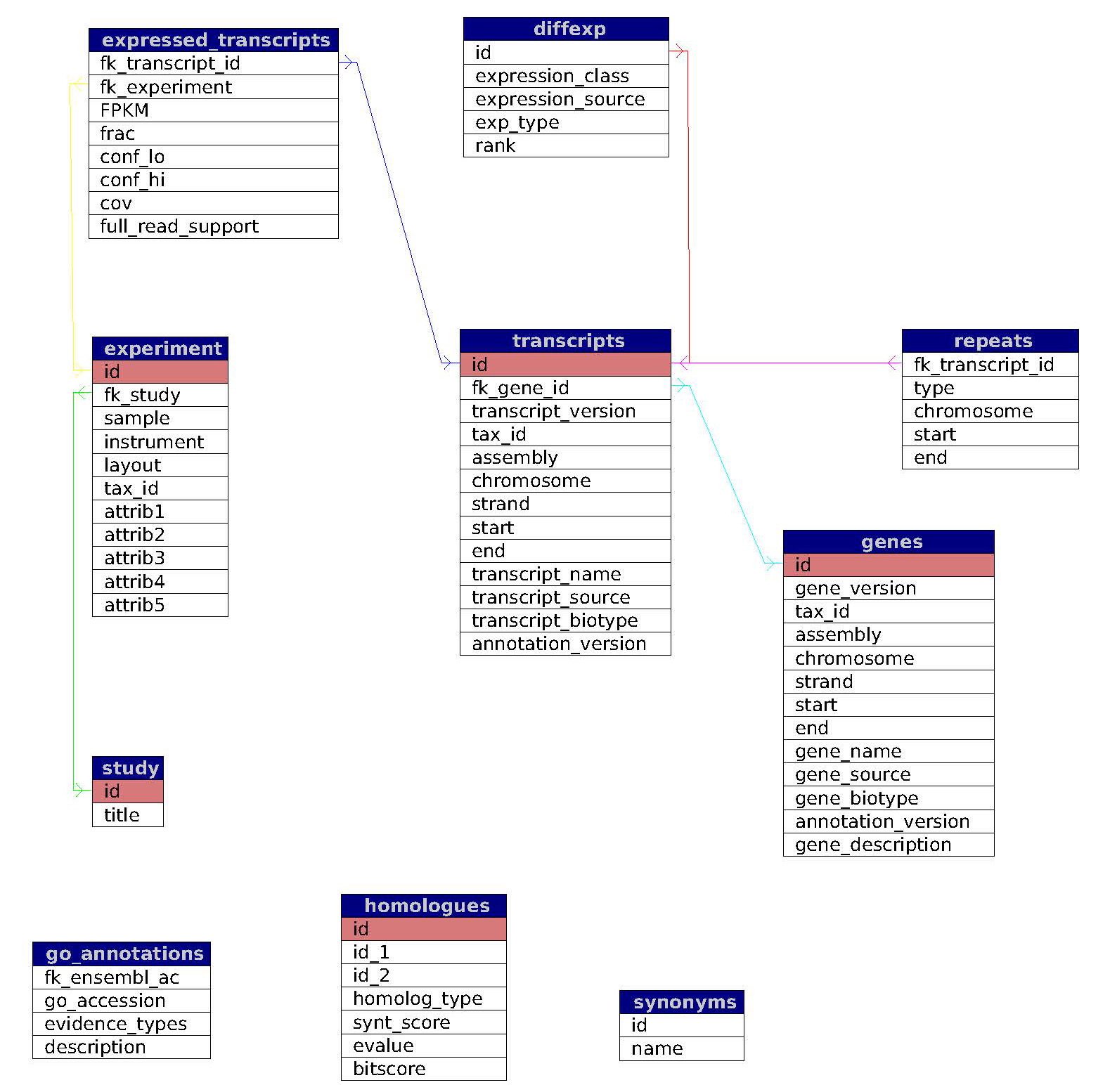

Supplement: Supplementary Dataset 1 [file srep32475-s2.zip › Figure_S1-NEW.jpg]
